# Boosting of Magnetic, Ferroelectric, Energy Storage Efficiency, and Piezoelectric Properties of Zn Intercalated SrBi_4_Ti_4_O_15_-Based Ceramics

**DOI:** 10.3390/ma15145057

**Published:** 2022-07-20

**Authors:** Nawishta Jabeen, Altaf Ur Rehman, Najam Ul Hassan, Muhammad Adnan Qaiser, Anum Zaidi, Muhammad Usman Khan, Imtiaz Ahmad Khan, Muhammad Nouman

**Affiliations:** 1 Department of Physics, Fatima Jinnah Women University, Rawalpindi 46000, Pakistan; anum.zaidi@fjwu.edu.pk; 2 Department of Physics, Riphah International University, Lahore 54000, Pakistan; altafqau1@gmail.com; 3 Department of Physics, Division of Science and Technology, University of Education, Lahore 54000, Pakistan; najam.hassan@ue.edu.pk; 4 College of Physics and Optoelectronic Engineering, Shenzhen University, Shenzhen 518060, China; ahmad.hussain@phys.uol.edu.pk; 5 Department of Physics, The University of Lahore, Sub-Campus Sargodha, Sargodha 40100, Pakistan; usman.khan@phys.uol.edu.pk (M.U.K.); nawishta.jabeen@phys.uol.edu.pk (I.A.K.); dr.ahmed.njust@gmail.com (M.N.)

**Keywords:** energy storage efficiency, SrBi_4_Ti_4_O_15_, ZnO, ferroelectric, piezoelectric, dielectric

## Abstract

An appropriate amount of Zn-ions are incorporated into the high Curie temperature bismuth layer-structure ferroelectric material to fabricate Sr_0.2_Na_0.4_Pr_0.4_Bi_4_Ti_4_O_15_:*xwt%*ZnO; (SNPBT:*x*Zn), with *x* = 0, 0.10, 0.15, and 0.20 ceramic series to investigate the magnetic, ferroelectric, and energy storage efficiency and piezoelectric properties. Pure SNPBT and SNPBT:*x*Zn ceramics have maintained their structure even after the intercalation of Zn-ions at the lattice sites of SNPBT. The addition of ZnO in SNPBT has improved the multifunctional properties of the material at *x* = 0.15. At room temperature, SNPBT:0.15Zn has shown a high relative density of 96%, exhibited weak ferromagnetic behavior along with a low saturation magnetization (*M_s_*) of 0.028 emu/g with a low coercive field of 306 Oe, a high remnant polarization (*P_r_*) of 9.04 µC/cm^2^, a recoverable energy density ($W_{rec}$) of ~0.5 J/cm^3^, an energy conversion efficiency (*η*) of ~41%, a high piezoelectric co-efficient (*d_33_*) of 21 pC/N, and an impedance of 1.98 × 10^7^ Ω, which are much improved as compared to pure SBT or pure SNPBT ceramics. Dielectric Constant (*ɛ_r_*) versus temperature plots present the sharp peak for SNPBT:0.15Zn ceramic at a Curie temperature (*T_C_*) ~ 605 °C, confirming the strong ferroelectric nature of the ceramic. Moreover, SNPBT:0.15Zn ceramic has shown strong, piezoelectric, thermally stable behavior, which remains at 76% (16 pC/N) of its initial value even after annealing at 500 °C. The achieved results clearly indicate that SNPBT:0.15Zn ceramic is a promising candidate for future wide-temperature pulse power applications and high-temperature piezoelectric devices.

## 1. Introduction

In the growing field of multifunctional materials, bismuth layered-structured ferroelectrics (BLSFs) have attained considerable attention due to their high dielectric constant (*ɛ*_r_), low dielectric loss (tan δ), and importantly high Curie temperatures (*T_C_*) [1,2]. BLSFs with high piezoelectric properties are suitable potential candidates to be utilized in sensors that can operate at high temperatures [3,4]. Moreover, the low aging rate due to their fatigue-free nature and strong anisotropic behavior has made these materials capable of being utilized in actuators, filters, and transformers [5]. In the modern era of technology, researchers have focused their attention towards the use of BLSFs in the development of the nonvolatile random access memory (FeRAM), where such materials are impressive in terms of saving the input information regardless of whether the power is turned off [6]. Lead-based and lead-free perovskite materials are highly investigated because of their high piezoelectric, high dielectric constant, low dielectric loss, and larger remnant polarization (*P_r_*), but due to the green issue and low operating temperature of lead-based perovskites, their usage has been restricted in several applications [7,8,9,10].

The (Bi_2_O_2_)^2+^ (A_m-1_B_m_O_3m+1_)^2−^ is a general formula for the Aurivillius family; the *A*-site can be occupied by monovalent, divalent, or trivalent ion, the *B*-site can be occupied by tetravalent, pentavalent, or hexavalent ions, and *m* = 2, 3, or 4 shows the number of perovskite layers separated by (Bi_2_O_2_)^2+^ layers [11,12]. Research on the pure BLSFs or modified BLSFs is mainly focused on achieving high piezoelectric behavior for high-temperature piezoelectric applications and also maintaining low dielectric loss, high resistivity, and good ferroelectric properties. It is observed that the properties of the BLSFs are strongly dependent on the number of perovskite units (A_m-1_B_m_O_3m+1_)^2−^, which can be controlled to great extent by doping the suitable dopant at *A* or *B*-sites [13,14]. Strontium bismuth titanate, or SrBi_4_Ti_4_O_15_ (SBT), with *m* = 4 ceramics is the widely studied material among the BLSFs family; it possesses the orthorhombic symmetry with *A_21_am* space group at room temperature, which transforms to tetragonal (*I*4*/mmm*) above transition temperature *T_C_* [14,15,16]. The suitable *A* and *B*-site dopants for SBT have recently shown interesting behaviors regarding the piezoelectric, ferroelectric, and pyroelectric properties. Recently, Wang et al. have studied the piezoelectric and dielectric properties of SBT ceramics with the addition of different oxides, where the modified Sr_0.92_Gd_0.053_Bi_4_Ti_4_O_15_ + 0.2 wt% Cr_2_O_3_ has shown the best merits with piezoelectric *d_33_* ~ 28 pC/N [17]. Ramana et al. have reported the high remnant polarization (*P_r_*) of ~9.2 µC/cm^2^ for the *A*-site doping of Pb in SBT (Sr_1−x_Pb_x_Bi_4_Ti_4_O_15_) ceramics [18]. Rajashekhar et al. have reported an improvement in the Curie temperature (*T_C_*) of ~623 °C for the modified Sr_0.2_Na_0.4_Pr_0.4_Bi_4_Ti_4_O_15_ ceramic as compared to *T_C_* ~ 535 °C of pure SBT ceramic [19]. Wang et al. have reported the thickness electromechanical coupling factor *k_t_* ~ 19.4% and planar electromechanical coupling factor *k_p_* ~ 3.5% at room temperature for the Co-modified SBT ceramics [20]. On the other hand, Zinc Oxide (ZnO) possesses unique characteristics, with good thermoelectric properties, high electron mobility, good corrosion resistance, thermal stability, and low toxicity [21,22]. Many functional materials with ZnO (dopant/additive) in the form of ceramics have become a strong topic of research in the field of sensors, actuators, optoelectronics, and the various types of energy converters into electrical energy [23]. Xu et al. have reported the enhancement in the dielectric properties of Zn-doped CaCu_3_Ti_4_O_12_ thin films [24]. Liu et al. have reported improvement in the ferroelectric properties (*P_r_* ~ 10 µC/cm^2^) of the 0.3% (mol%) doping of Zn in Ba_0.9_Sr_0.1_TiO_3_ ceramics because Zn is a kind of polar molecule and its doping into the ceramic possesses the property to make the spontaneous polarization easier [25]. Moreover, Zhang et al. have reported the reduced back-switching of aligned domains in Bi_0.5_Na_0.5_TiO_3_:ZnO composite so that the composite can retain the piezoelectric coefficient for higher temperature as compared to pure Bi_0.5_Na_0.5_TiO_3_ ceramic [26].

Herein, a SrBi_4_Ti_4_O_15_-based ceramic series is fabricated with the addition of ZnO content in the form of Sr_0.2_Na_0.4_Pr_0.4_Bi_4_Ti_4_O_15_:*xwt%*ZnO; (SNPBT:*x*Zn) (with *x* = 0, 0.10, 0.15 0.20) to investigate the structural as well as morphological effects. Ceramic with low Zn content SNPBT:0.15Zn has shown weak ferromagnetic and strong ferroelectric (*P_r_* ~ 9.04 µC/cm^2^) behaviors, along with an improved piezoelectric coefficient (*d_33_* ~ 21 pC/N) as compared to pure SBT or SNPBT ceramics.

## 2. Fabrication and Characterization

SrBi_4_Ti_4_O_15_ (SBT) and Sr_0.2_Na_0.4_Pr_0.4_Bi_4_Ti_4_O_15_ (SNPBT) compositions were prepared by the conventional solid state reaction method, with a rapid increase/decrease of temperature > 50 °C/min. Commercially available (Sigma Aldrich with 99.9% purity), raw powders of SrCO_3_, Bi_2_O_3_, Na_2_CO_3_, Pr_2_O_3_, and TiO_2_ were weighed according to the stoichiometric ratio of pure SBT and SNPBT, mixed by milling machine for 24 h in ethanol, and dried at 100 °C overnight. Dried powders were calcinated at 850 °C for 4 h, ground, and milled again using the same above parameters. Circular pellet disks with a diameter of 11 mm were prepared for pure SBT powder under the cold isostatic pressure of 100 MPa at room temperature and sintered at 1100 °C for 4 h with the rapid increase/decrease of temperature > 50 °C/min, while ZnO (99.9%) was added in the calcinated single-phase SNPBT powder with 0.00 wt% to 0.20 wt% (i.e., *x* = 0, 0.05, 0.10, 0.15, 0.20 wt%) variation. Afterwards, the powders were pelletized and sintered at 1100 °C for 4 h with a rapid increase/decrease of temperature > 50 °C/min.

Structural analysis of the material was carried out by X-ray diffraction study using XRD, PANalytical, Netherlands, 40 kV, 30 mA, *Cu-Kα* 1, *λ* = 1.54056 *Å*, step: 0.02° at room temperature and 2θ ranging 15–70°; later, the simulation of these XRD patterns was performed by the Rietveld method using Material Studio. A field emission scanning electron microscope (FE-SEM, FEI Quanta 200, Hillsboro, OR, USA) was employed to observe the microstructure and elemental distribution of the ceramic material. Further, ceramics were polished to 0.7 to 0.9 mm thickness, and for the analysis of ferroelectric, dielectric, and electrical properties, Ag electrodes were coated on both surfaces of the polished ceramics. Ferroelectric analysis (*P-E* loops) was determined at 1 Hz using a ferroelectric analyzer (aixACCT TF Analyzer 2000; Germany) at room temperature. Afterwards, at 120 °C pure SBT, SNPBT, and SNPBT:*x*Zn ceramics were fully polarized in the silicon oil bath under a high *DC* electric field of 150 kV/cm, and the piezoelectric *d_33_* coefficient was measured at 60 Hz by using a piezo-*d_33_* meter (IAAS ZJ-30, Institute of Acoustics of CAS, Beijing, China). For the observance of thermal stability of piezoelectric properties of the ceramic material, fully polarized ceramics were annealed at a specific temperature range (20–600 °C) for 30 min; then, after annealing *d_33_* values were measured at room temperature. Impedance, relative dielectric constant (*ε_r_*), and dielectric loss (*tanδ*) as a function of frequency were measured by Agilent 4294A impedance analyzer. Dielectric permittivity (*ε_r_*) and dielectric loss (*tanδ*) as a function of the temperature were analyzed by an LCR analyzer (HP4980A, Agilent, Santa Clara, CA, USA) attached to a programmable furnace. Magnetization measurements were taken by a vibrating sample magnetometer (Lake Shore 7404) at room temperature.

## 3. Results and Discussion

**Figure 1:**
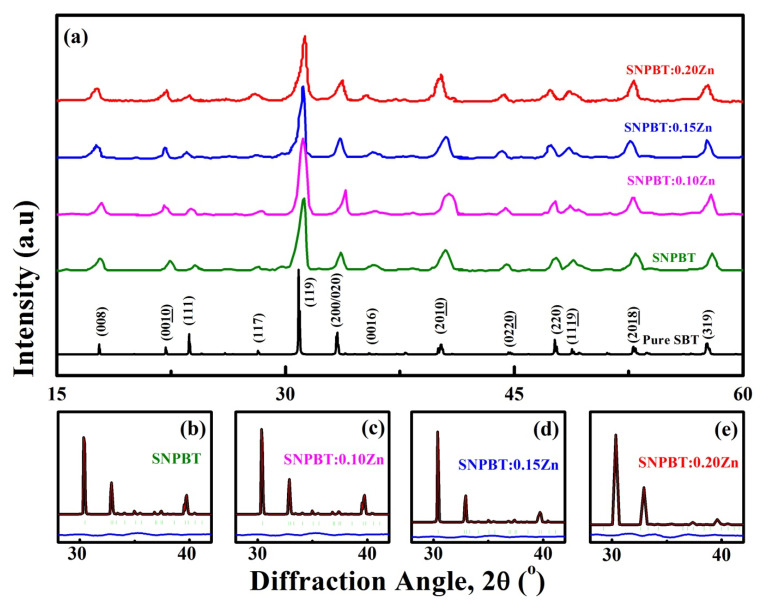
(**a**) Room temperature XRD patterns of pure SBT ceramics (bottom) and SNPBT:*x*Zn (*x* = 0–0.20) ceramics, Rietveld refinement results of the (**b**) Pure SNPBT, (**c**) SNPBT:0.10Zn, (**d**) SNPBT:0.15Zn and (**e**) SNPBT:0.20Zn.

In Figure 1a, a XRD comparison of pure SBT, pure SNPBT, and SNPBT:*x*Zn (*x* = 0.10, 0.15, 0.20) ceramics is illustrated to optimize the effect of the Zn^2+^ addition in the reported SNPBT. It is evident from Figure 1 that all ceramic samples have maintained their single orthorhombic phase, with *A2_1_am* space group structure and patterns matching well with the standard PDF Card # 43-0973 [19,27]. All XRD patterns have not shown any impurity or extra peaks, indicating that the additive Zn-ions have been incorporated into the crystal lattice of SNPBT. (119) is the strongly intense diffraction peak of pure SBT (for *m* = 4 of general BLSF formula) around the diffraction angle of 30^o^, which is known to occur at (112*m +* 1) [28,29]. A similar trend has been observed for the pure SNPBT and SNPBT:*x*Zn ceramics. It is clear from the XRD analysis that the addition of Zn^2+^ for Sr^2+^ and Bi^3+^ has not shown a strong impact on the phase formation, but a slight shifting of the maximum intense (119) peak towards the higher angle is observed. The type of compressive stress influenced by the additive Zn^2+^ ions (with lower ionic radii) has not affected the lattice parameters of SNPBT symmetry (with larger ionic radii Sr^2+^, Na^+^, Bi^3+^) [30,31], which is only possible if a lesser amount of the additive has been incorporated in the lattice site. Herein, it can be concluded that the additive (lesser content) has been incorporated successfully into the lattice without damaging the crystal structure. Higher substitutions usually result in heavy lattice distortions and occasional local phase formations or even the change of the space group. However, here it is not the case, as the ceramics have maintained their structure. The XRD patterns of SNPBT:*x*Zn (*x* = 0, 0.1, 0.15, 0.20) ceramics were simulated with the Rietveld method by using Material Studio (Figure 1b–e). A high symmetric orthorhombic *A2_1_am* space group structure was treated as the reference structure for the simulation. Two standard parameters of the Rietveld simulation are *R_wp_* and *R_p_*, which remained less than 6% and 4% for all simulated samples, confirming that ceramics have maintained their structure even after the incorporation of the Zn^2+^ ions at the lattice sites of SNPBT.

**Figure 2:**
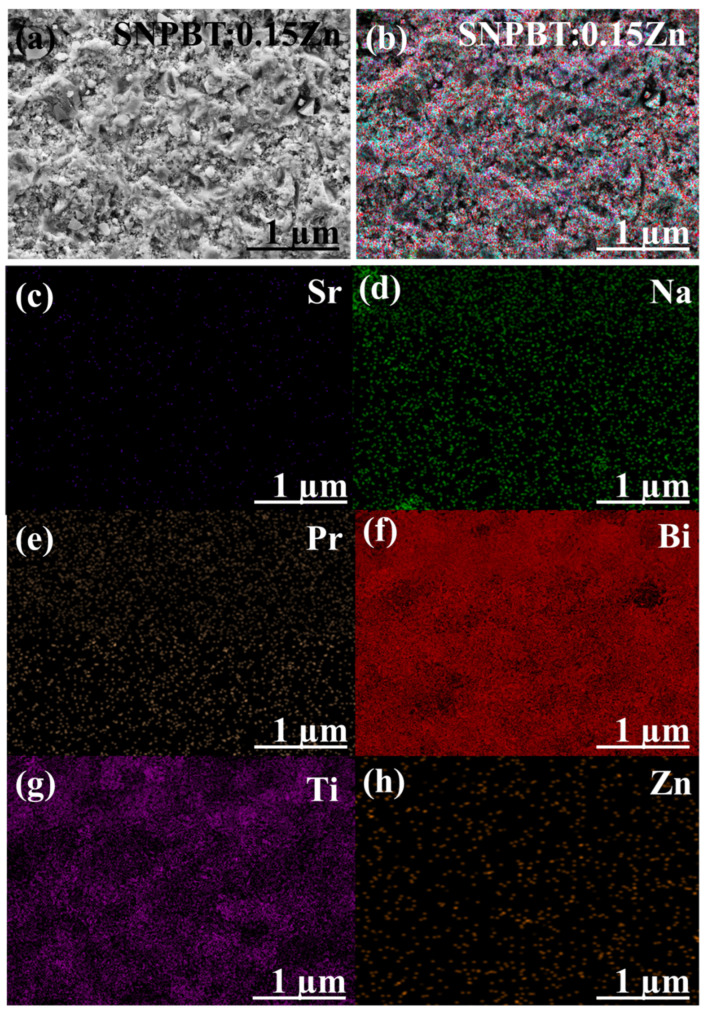
(**a**) FE-SEM micrograph of SNPBT:0.15Zn ceramic, Colored elemental mapping with precise compositional analysis of (**b**) SNPBT:0.15Zn; Dispersion analysis of elements (**c**) Sr, (**d**) Na, (**e**) Pr (**f**) Bi, (**g**) Ti, and (**h**) Zn.

The visual compositional elemental analysis of typical 2D elemental mapping is shown in Figure 2a–h. Here, an SEM image of SNPBT:0.15Zn ceramic is presented in Figure 2a, where uniformity of the grain size 1–2 μm is observed. This refinement of the grain size has been achieved due to the addition of Zn-ions at the A-site of SNPBT lattice, which has not only reduced the lattice diffusivity [32] but also resulted in no visible holes or cracks in the ceramic. The combined elemental distribution of all involving elements of SNPBT:0.15Zn is shown in Figure 2b. In Figure 2c,d, a high degree of dispersion in the 2D elemental mapping of Bi and Ti is visible, depending upon their high stoichiometric ratios in SNPBT:0.15Zn ceramic. On the contrary, a lower dispersion degree of Sr, Na, Pr and Zn (Figure 2e,h) endorses the lower stoichiometric ratio of the elements in the SNPBT:0.15Zn chemical formula. Figure 2h shows that Zn^2+^-ions are uniformly distributed throughout the ceramic, which is a clear indication of the incorporation of the Zn^2+^-ions at the lattice sites of the SNPBT host. In other words, Zn^2+^-ions have segregated at the grain boundaries of SPPBT. The high relative density of SNPBT:0.15Zn ceramic is ~96%, which points out that the addition of Zn has overcome the vacancies created by the evaporation of Bi components at high-temperature fabrication and made the sample compact for thermal and electrical measurements.

**Figure 3:**
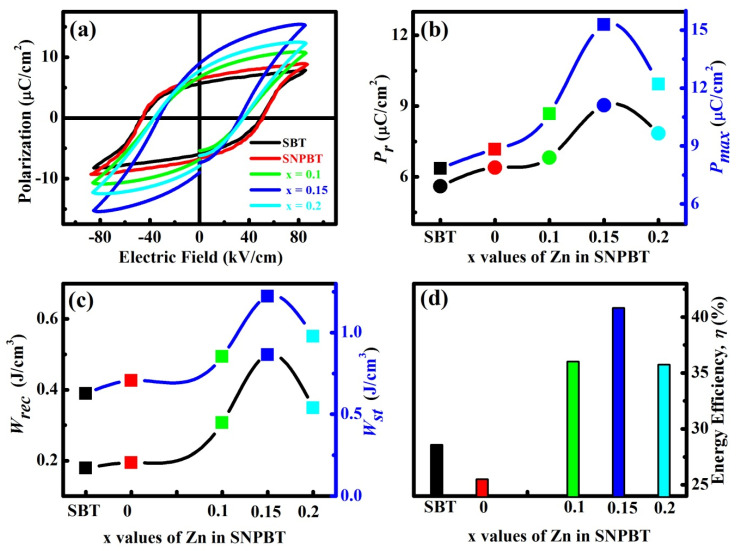
(**a**) *P-E* loops of pure SBT and SNPBT:*x*Zn (*x* = 0–0.2) ceramics, (**b**) *P_r_* and *P_max_* of pure SBT and as a function of *x* value of SNPBT:*x*Zn ceramics, (**c**) *W_st_*, and *W_rec_* of the pure SBT and as a function of *x* value of SNPBT:*x*Zn ceramics. (**d**) Energy conversion efficiency (*η*) of the pure SBT and as a function of *x* value of SNPBT:*x*Zn ceramics.

Figure 3a is the representation of the polarization versus applied electric field (*P-E* loops) for the pure SBT, pure SNPBT, and SNPBT:*x*Zn (*x* = 0, 0.10, 0.15, and 0.20) ceramics, measured at room temperature and 1 Hz frequency. The saturated loops of SNPBT:*x*Zn (*x* = 0, 0.10, 0.15 and 0.20) ceramics are attained at the electric field of 80 kV/cm. Herein, SNPBT:0.15Zn ceramic has shown the highest remnant polarization (*P_r_* ~ 9.04 µC/cm^2^) and maximum polarization (*P_max_* ~ 15.28 µC/cm^2^) as compared to pure SBT (*P_r_* ~ 5.6 µC/cm^2^) and pure SNPBT (*P_r_* ~ 6.4 µC/cm^2^) ceramics. From this result, it is clear that *P_r_* is increasing with varying Zn concentration (Figure 3b). This can be accredited to the smaller cation (Zn) addition for Bi ions in the present layered perovskite compound. Throughout the sintering method, due to the volatile nature, bismuth vacancies will unavoidably appear, and at the same time, numbers of oxygen vacancies are created in order to meet the charge neutrality condition. The reduction of bismuth and oxygen vacancies due to the addition of Zn^2+^-ions and low defect mobility [31,33] are the main reasons for the increments in the ferroelectric property. Afterwards, for SNPBT:0.20Zn ceramic, the *P_max_* and *P_r_* values have reduced to 12.2 µC/cm^2^ and 7.84 µC/cm^2^, respectively, because a high content of Zn^2+^-ions has started to settle at the A-site of SNPBT host, resulting in overcoming the percolation threshold value. Now, the host SNPBT has started to accommodate the excessive Zn^2+^ ions at the grain boundaries resulting in a decrement in the ferroelectric behavior [1,9]. The comparison study of *P_max_* and *P_r_* values for the pure SBT, pure SNPBT and SNPBT:xZn (x = 0.10, 0.15, and 0.20) are shown in Figure 3b.

Dielectric capacitors have drawn considerable attention in the pulse power equipment field due to their highpower density, fast charge–discharge speed, and excellent chemical stability. The stored energy density ($W_{st}$), recoverable energy density ($W_{rec}$), and energy conversion efficiency (*η*) results are dependent on the measurements of *P-E* loops; their relations can be expressed as follows [34]:

$$W_{st}=\int_{0}^{Pmax}Edp$$

$$W_{rec}=\int_{Pr}^{Pmax}Edp$$

$$\eta =\frac{W_{rec}}{W_{st}}\times 100$$

where *P_max_*, *P_r_*, and *E* are the maximum polarization, remanent polarization, and electric field, respectively [35]. In Figure 3b,c, the energy storage properties (*W_st_*, *W_rec_*, and η) are calculated and plotted versus the *x* values of Zn in SNPBT at the applied electric field of 80 kV/cm. The *W_rec_* of all the samples is above 0.2 J/cm^3^ (0.2–0.5 J/cm^3^) and the efficiency is above 25% (25–41%). The highest *W_rec_* of 0.5 J/cm^3^ with high energy efficiency (41%) is achieved in the SNPBT:0.15Zn ceramic.

**Figure 4:**
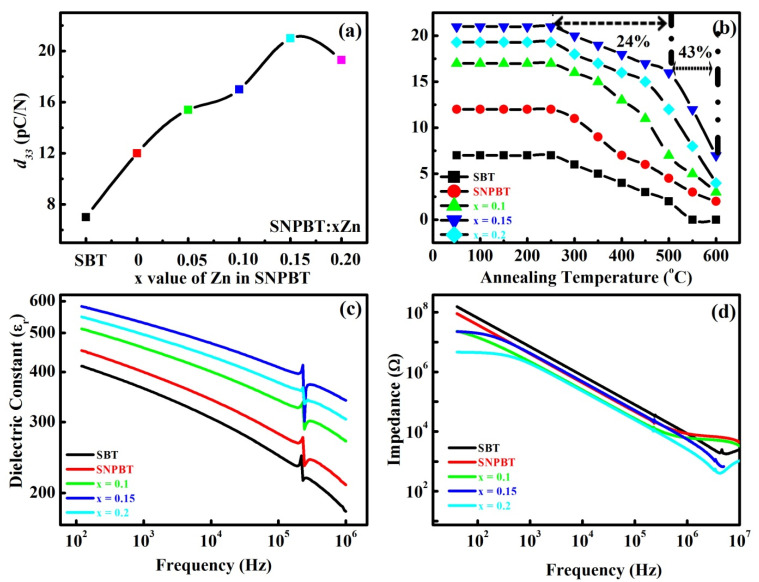
(**a**) Piezoelectric coefficient (*d_33_*) measurement of pure SBT and SNPBT:*x*Zn (*x* = 0–0.20) ceramics as a function of xwt%ZnO addition in SNPBT, samples (**b**) *d_33_* of the poled pure SBT and SNPBT:*x*Zn ceramics, annealed at 28–600 °C, (**c**) Dielectric constant (*ε_r_*) versus frequency graphs of poled pure SBT and SNPBT:*x*Zn (*x* = 0–0.20) ceramics, (**d**) Impedance versus frequency analysis of poled pure SBT and SNPBT:*x*Zn (*x* = 0–0.20) ceramics at room temperature.

The maximum value of the piezoelectric coefficient (*d_33_ ~* 21 pC/N) has been achieved in the SNPBT:0.15Zn ceramic sample as compared to pure SBT (7 pC/N) and SNPBT (12 pC/N) (Figure 4a). Figure 4a is the piezoelectric coefficient (*d_33_*) versus *x*wt%Zn addition in SNPBT and pure SBT (for comparison) measurements taken at room temperature. All the ceramic samples were polarized at 120 °C under a high *DC* electric field of 150 kV/cm. To further confirm the thermal stability of the *d_33_* value, the degradation of polarization in the ceramics is observed after annealing the samples at different temperature ranges (20–600 °C) for 30 min; the retained *d_33_* at room temperature is depicted in Figure 4b. The *d_33_* value of pure SBT has reduced from 7 pC/N (room temperature) to 2 pC/N (at 500 °C) due to the well-known thermal depoling effect. Similarly, the value of *d_33_* for pure SNPBT has remained strong at 5 pC/N at 500 °C due to the high Curie-temperature (623 °C) of the ceramic [19]. However, the piezoelectric coefficient of SNPBT:0.15Zn ceramic is the most stable, with a 24% decrement in the *d_33_* value at 500 °C (16 pC/N) as compared to its room temperature value (21 pC/N). During the annealing process, ceramics transit from an aligned domain structure to a randomized domain structure is a natural phenomenon. When the external poling field is removed, the depolarization field tends to perform the complete back-switching of the domains in the ceramics at the diffusion temperature. During the poling process, the applied external poling field was the reason for creating the alignment of ferroelectric domains in pure SNPBT grains and unbound misfit compensation charges at SNPBT/SNPBT grain boundaries. The additive ZnO particles (naturally semiconducting) have occupied those unbound misfit places, which has resulted in the further growth of mobile electronic charge distribution over the SNPBT grain boundaries. So, the depolarization field, to some extent, is compensated by the charges introduced by ZnO. Consequently, after the removal of the external poling field, SNPBT grains have maintained their poled states and will reduce the back-switching effect. Insofar as the behavior of ZnO is important, it is considered that ZnO will contribute its semiconductor nature to ensure that it will behave as a charge pool, so as to reduce the piezoelectric degradation behavior [36]. The value of *d_33_* will start to reduce for SNPBT:0.20Zn ceramic (19.3 pC/N), as the solubility limit of Zn in SNPBT has reached (Percolation threshold); afterward, Zn ions will start to gather at the grain boundaries of SNPBT counterpart, affect the piezoelectric degradation, and produce the internal bias field [1,9]. In such a case, the excess of Zn-ions with a semiconductor nature will be the reason for high conductivity and the degradation of *d_33_*.

The dielectric constant (*ɛ_r_*) versus frequency graph of fully poled pure SBT, pure SNPBT, and SNPBT:*x*Zn (*x* = 0.1, 0.15 and 0.2) ceramics is presented in Figure 4c, measured at room temperature. In this, the ceramic SNPBT:0.15Zn has shown the best dielectric constant merits with *ɛ_r_* ~ 576 at a frequency of 100 kHz, which is higher than pure SBT (*ɛ_r_* ~ 412) and pure SNPBT ceramics (*ɛ_r_* ~ 451). As all the ceramic samples were fully poled, the dielectric anomalies along the thickness of the ceramic will appear at a certain frequency (resonance/anti-resonance frequency ~ 230/243 kHz). Poled ceramic samples have the ability to start deforming their piezoelectric effect under the applied 0.5 AC voltage, which results in the creation of dielectric anomalies. The dependence of impedance on the frequency plots of fully polarized SBT, SNPBT, and SNPBT:*x*Zn (*x* = 0.1, 0.15 and 0.2) ceramic samples is presented in Figure 4d. The ceramic sample SNPBT:0.15Zn has displayed the high impedance of ~ 1.98 × 10^7^ Ω at room temperature at about a frequency of 100 Hz, which is much consistent with the impedance of pure SBT (6.4 × 10^7^ Ω) and SNPBT (3.7 × 10^7^ Ω) ceramics at 100 Hz. This clearly demonstrates that samples have maintained their resistive behavior, which is the trademark property of the BLSFs. The impedance drop behavior with the variation of frequency is similar for all the ceramic samples. Moreover, the impedance/dielectric anomalies are also present in the impedance versus frequency measurements as all samples were fully poled, so deformation and vibration along the thickness at a certain frequency (similar as in the case of *ɛ_r_* vs. frequency plot) are observed due to the piezoelectric (*d_33_*) effect [10].

**Figure 5:**
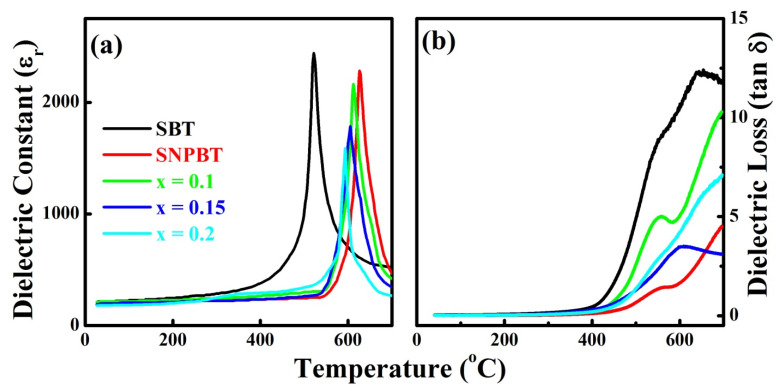
(**a**) The dependence of relative dielectric constant (*ε_r_*) as a function of temperature for pure SBT and SNPBT:*x*Zn (*x* = 0–0.20) ceramics, measured at 100 kHz. (**b**) Dielectric loss (*tanδ*) as a function of temperature for pure SBT and SNPBT:*x*Zn (*x* = 0–0.20) ceramics.

The thermal stability of the high piezoelectric coefficient in materials can direct the sensitivity of the device to be utilized in high-temperature piezoelectric devices. Similarly, the thermal stability of *d*_33_ will not only describe the strength of the material but also the efficiency of the material under applied high temperatures. The Curie temperature (*T_C_*) of materials plays a vital role, as materials lose their complete piezoelectric and ferroelectric properties at a specific *T_C_.* The dielectric constant (*ɛ_r_*) versus temperature plots of pure SBT, SNPBT, and SNPBT:*x*Zn ceramics (*x* = 0.1, 0.15, and 0.2) are shown in Figure 5a for the temperature range of 0–700 °C, measured for the frequency 100 kHz, where pure SBT has shown the highest *ɛ*_r_ ~ 2320 at the *T*_C_ ~ 521 °C, consistent with previous reports [37]. The pure SNPBT ceramic has shown the *ɛ_r_* ~ 2205 at the highest *T_C_* of ~ 626 °C, which is similar to already reported information [19]. The addition of small content Zn^2+^-ions in the SNPBT counterpart (SNPBT:*x*Zn) has resulted in little degradation in the values of *ɛ_r_* and *T_C_*, as compared to pure SNPBT. SNPBT:0.10Zn, SNPBT:0.15Zn, and SNPBT:0.2Zn have given the *ɛ_r_* values of 2160, 1786, and 1580 at the *T_C_* values of 612 °C, 605 °C, and 593 °C, respectively. Here, it is notable that SNPBT:*x*Zn (*x* = 0.10, 0.15, 0.20) ceramics have shown a high *T_C_,* even much higher than pure SBT, which is the specific reason for getting the high *d*_33_ values of the ceramics even after 500 °C. The achieved result clearly demonstrates the importance of the material to be utilized in multifunctional devices at such high temperatures. Dielectric loss versus frequency graphs of pure SBT, SNPBT, and SNPBT:*x*Zn ceramics (*x* = 0.1, 0.15, and 0.2) are plotted in Figure 5b, which shows the very low values of dielectric loss for all ceramic samples even at the high temperature of >500 °C, which is an indication that the Zn-ions have successfully incorporated at the lattice sites of the structure without creating any destruction. Second, Zn-ions have also occupied the vacancies, which were generated by the evaporation of Bi or oxygen counterparts, confirming the strength and compactness of the ceramics for such a high temperature and high frequency.

**Figure 6:**
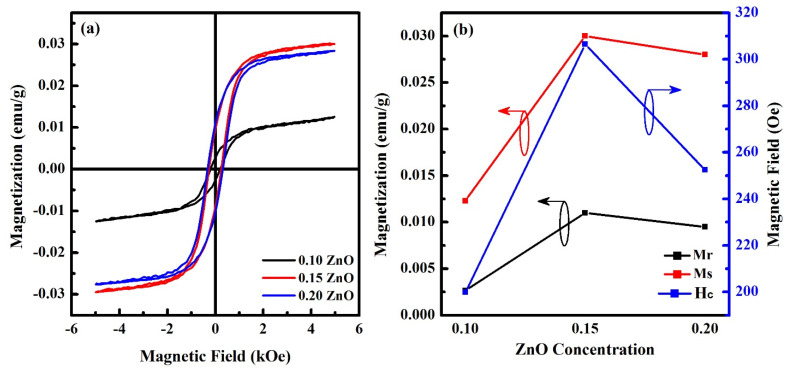
(**a**) Magnetization versus magnetic field loops of SNPBT:*x*Zn (*x* = 0.10–0.20) ceramics. (**b**) Magnetization dependent on ZnO concentration in SNPBT.

In Figure 6, the magnetization versus magnetic field measurements of SNPBT:*x*Zn (*x* = 0.10–0.20) ceramics are taken at room temperature and show the weak ferromagnetic behavior. The increment in saturation magnetization was seen as the ZnO concentration was increased, indicating that the addition of ZnO to the SNPBT ceramics improves their ferromagnetic behavior. The highest saturation magnetization (*Ms*) of 0.028 emu/g with a low coercive field of 306 Oe was observed for SNPBT:0.15Zn. The weak ferromagnetism is related to the presence of the magnetic interactions of ZnO and uncompensated magnetic spin configurations in SNPBT ceramics. Furthermore, as described earlier in the SEM analysis (Figure 2h), Zn^2+^-ions are uniformly distributed throughout the ceramic surface, and few are segregated at the SNPBT/SNPBT grain boundaries, which is the reason for generating a huge number of uncompensated surface spins, resulting in a long-range ferromagnetic order [38].

## 4. Conclusions

In this work, a high Curie temperature SBT-based SNPBT:*x*Zn with *x* = 0–0.20 ceramic series is fabricated to examine the effect of Zn-ions intercalation in the lattice sites of SNPBT materials to investigate the multifunctional properties of ceramics. All ceramics have maintained their crystal structure. The SNPBT:0.15Zn ceramic has shown the pronounced properties that were much improved compared to pure SBT, pure SNPBT, and other ceramics. The SNPBT:0.15Zn ceramic has presented a high relative density of 96%, a high remnant polarization (*P_r_*) of 9.04 µC/cm^2^, a recoverable energy density ($W_{rec}$) of ~0.5 J/cm^3^, an energy conversion efficiency (*η*) of ~41%, a high piezoelectric coefficient (*d_33_*) of 21 pC/N, and an impedance of 1.98 × 10^7^ Ω at room temperature. The high Curie temperature (605 °C) of ceramic is the main reason to achieve a thermally stable piezoelectric response (16 pC/N) with just a 24% drop at 500 °C from its initial value. The above stated properties prove the worth of the material to be utilized for high-temperature piezoelectric devices.
